# Aspirin and multiple sclerosis

**DOI:** 10.1186/s12916-015-0394-4

**Published:** 2015-06-29

**Authors:** Sheila Tsau, Mitchell R. Emerson, Sharon G. Lynch, Steven M. LeVine

**Affiliations:** 1 grid.412016.00000000121776375Department of Molecular and Integrative Physiology, University of Kansas Medical Center, Kansas City, KS USA; 2 grid.260024.2Department of Pharmaceutical Sciences, College of Pharmacy-Glendale, Midwestern University, Glendale, AZ USA; 3 grid.412016.00000000121776375Department of Neurology, University of Kansas Medical Center, Kansas City, KS USA

**Keywords:** Antiphospholipid antibodies, Aspirin, Experimental autoimmune encephalomyelitis, Fatigue, Multiple sclerosis, Salicylate, Stroke, Thrombosis

## Abstract

Aspirin is widely used to lessen the risks of cardiovascular events. Some studies suggest that patients with multiple sclerosis have an increased risk for some cardiovascular events, for example, venous thromboembolism and perhaps ischemic strokes, raising the possibility that aspirin could lessen these increased risks in this population or subgroups (patients with limited mobility and/or antiphospholipid antibodies). However, aspirin causes a small increased risk of hemorrhagic stroke, which is a concern as it could potentially worsen a compromised blood-brain barrier. Aspirin has the potential to ameliorate the disease process in multiple sclerosis (for example, by limiting some components of inflammation), but aspirin also has the potential to inhibit mitochondrial complex I activity, which is already reduced in multiple sclerosis. In an experimental setting of a cerebral ischemic lesion, aspirin promoted the proliferation and/or differentiation of oligodendrocyte precursors, raising the possibility that aspirin could facilitate remyelination efforts in multiple sclerosis. Other actions by aspirin may lead to small improvements of some symptoms (for example, lessening fatigue). Here we consider potential benefits and risks of aspirin usage by patients with multiple sclerosis.

## Introduction

Multiple sclerosis (MS) is a debilitating chronic disease characterized by inflammation, demyelination, axonal transection, and neurodegeneration in the central nervous system (CNS), leading to motor, sensory, and cognitive difficulties [1, 2]. Although MS is thought to have an autoimmune component, other mechanisms contribute to disease progression, for example, mitochondrial dysfunction, activated microglia, and intracerebral vascular changes [3–6]. Vascular changes include blood-brain barrier (BBB) leakage [7, 8], areas of decreased or increased cerebral perfusion [9–13], and vessel occlusion [14–16]. In line with these vascular changes, some reports have suggested that patients with MS have a greater risk of ischemic stroke and venous thrombosis [17–23]. These patients also have a higher incidence of antiphospholipid antibodies (APLAs), the main feature of antiphospholipid syndrome (APS); and patients with APS have an elevated risk of ischemic stroke and thrombosis.

Aspirin (acetylsalicylic acid, ASA) is a popular and readily available drug that has a variety of effects including alleviating pain and reducing fever, and it is often used for the secondary prevention of cardiovascular events in patients at risk [24–26]. Given the elevated risks for stroke and venous thrombosis in MS, ASA may help counter the development of these conditions [27–29]. ASA may also positively impact other facets of MS disease activity (for example, it may reduce inflammation, lessen fatigue, and promote remyelination). However, ASA usage increases the risk for hemorrhagic stroke [30–32], indicating the possibility that ASA could worsen BBB disruption in MS. ASA also has the potential to interfere with mitochondrial complex I activity [33], which has reduced activity in MS [34, 35]. Thus, the risk-to-benefit ratio for ASA usage by MS patients is unclear. This paper will review the potential benefits (Table 1) and risks (Table 2) associated with ASA usage in patients with MS.

**Table 1:** Potential benefits of ASA usage in MS

Feature	Description	ASA’s effect
Ischemic stroke	MS patients may have an increased risk of stroke [17, 18, 20, 36, 37].	ASA reduces the risk of ischemic stroke in some subjects, for example, those who had a previous stroke [24–26, 79]. ASA decreases platelet activation and aggregation through irreversible inhibition of platelet COX-1, and the resultant decrease in TXA_2_ production has a cardioprotective effect [57, 64]. It is unknown whether the risk of strokes in MS patients will be reduced in response to ASA.
Thrombosis	MS patients have an increased risk of venous thrombosis [18, 19, 21–23, 39].	ASA lowers the incidence of venous thrombosis in some subjects, for example, orthopedic surgery patients and those who experienced an unprovoked venous thromboembolism [27, 28, 88]. It is not known whether the risk of venous thrombosis is reduced in response to ASA in MS patients, but ASA reduces the risk for first thrombosis in patients with APLAs [107].
	A higher percentage of MS patients have APLAs than controls [96–98]. APLAs are a diagnostic feature of APS, which involves thromboses.	
Platelets	Platelets are activated in MS and have been implicated in contributing to MS pathogenesis, such as by promoting inflammation [71, 72, 111].	Anticoagulants decreased the severity of EAE [110, 211]. ASA lowers one indicator of platelet activation in MS patients.
Fibrin	Limiting fibrin formation reduced EAE disease activity [114, 115]. Fibrin deposition may activate microglia [113].	ASA may lessen fibrin deposition and induce fibrinolysis [116].
Thrombin	Is thought to promote inflammatory disease states of the CNS [117], and thrombin is associated with multiple pathological features in EAE [118].	ASA may decrease thrombin at microvascular injury sites [119].
Microglia	Activated microglia can have a pro-pathogenic role in MS [3–5].	ASA may reduce production of proinflammatory cytokines and reactive oxygen species (ROS) by microglia [155–157].
Inflammation	Multiple components of inflammation (for example, ROS, proinflammatory cytokines) are thought to contribute to MS pathogenesis.	ASA may promote the resolution of inflammation via the production of lipoxin A_4_ [158, 159].
Remyelination	Remyelination is incomplete in MS [212].	ASA may increase ciliary neurotrophic factor and promote the differentiation and proliferation of oligodendrocyte precursors [161, 163].
Fatigue	Fatigue is a common symptom of MS.	ASA may reduce fatigue in MS patients via antipyretic effects or by countering proinflammatory cytokines [164, 176, 177].
Depression	Depression is more common in MS than in the general population [180].	ASA usage may lower the risk for major depression, and some evidence shows that ASA in combination with fluoxetine enhances treatment for depression [181, 182, 188]. It is unknown whether ASA would help to reduce depression in MS, but other studies suggest that it can have negative impacts or side effects in depressed patients (see Table 2)
General disease activity	MS patients given calcium aspirin (Solprin) [147, 148] or EAE subjects given sodium salicylate or ASA [149–152]. Studies were performed decades ago.	Overall, the outcome is inconclusive. There was no effect in MS patients [147, 148], but evaluation was done using an outdated measure of disease activity. In EAE, disease onset was delayed and/or disease incidence reduced in 3 out of 4 studies [149, 151, 152]. Treatment after clinical signs appeared resulted in no benefit [149], and in one study disease severity was increased although disease onset was delayed [151].

**Table 2:** Potential risks of aspirin usage in MS

Adverse event	Description	ASA’s mechanism
Cerebral bleeding/hemorrhagic strokes)	There is an increase in the incidence of intracranial hemorrhages with antiplatelet treatment [30–32, 79]. Because MS patients can have a compromised BBB [7, 8], the risk of intracerebral bleeding in MS may be greater with ASA usage.	Platelets/coagulation may act to limit BBB damage in MS. ASA’s antiplatelet/anticoagulation properties potentially increase the likelihood that BBB leakage could be prolonged or worsened.
Mitochondrial function complex I inhibition	Mitochondrial complex I activity is reduced in MS [34, 35]. ASA may inhibit complex I [33], and the effect may be more pronounced with glutathione depletion [140], which is thought to occur in MS [141, 142]. Inhibition of complex I may lead to an increase in ROS production [143].	Direct inhibition of complex I by ASA [33].
Increased gastrointestinal bleeding	ASA treatment has been found to increase the incidence of gastrointestinal and other extracranial bleeding [31], and risk increases with patient age [198].	Anticoagulation (antiplatelet); ASA blocks the synthesis of gastroprotective prostaglandins via inhibition of COX-1, which increases gastrointestinal bleeding [198].
Increased risk of bleeding with concurrent use of ASA with an antidepressant (SSRI)	Both ASA and the antidepressant class selective serotonin reuptake inhibitors (SSRIs) increase risk of bleeding by themselves. Following an acute myocardial infarction, the combination of ASA and SSRI treatment increases the risk of bleeding in patients compared to ASA alone [191].	Anticoagulation (antiplatelet)
Adverse effects relative to depression	Concurrent use of ASA with citalopram may lead to an increased risk of adverse events, such as anxiety, akathesia, and suicidal behavior [189]. Also, NSAIDs may interfere with the antidepressant effects of SSRIs, preventing depressed patients from achieving relief [190].	The mechanism is unknown, but possibly NSAIDs are blocking the production of a protective mediator, for example, anti-inflammatory cytokine [189].
Hearing loss and tinnitus	Although rare, hearing loss and tinnitus have been reported with high dosages of salicylate (6–8 g or more per day) [200, 203, 204]. In men, regular usage of ASA also has been found to heighten the risk for loss of hearing, and the effect is more pronounced in younger individuals [213].	The mechanism is not known, but may be through suppressing GABAergic inhibition [203, 204]; sodium salicylate also affects cochlear function [200, 204]
Respiratory attacks, asthma	Approximately 10 % [207] to 21 % [206] prevalence of ASA-induced asthma in adult asthmatic patients and 5 % prevalence in asthmatic children, based on both retrospective/self-report studies and provocation studies [206].	COX enzyme inhibition leading to decreased PGE_2_ and enhanced leukotriene and histamine production [198, 206, 210].

## Cardiovascular risks in MS

### Patients with MS may have an increased risk for cardiovascular disease

Multiple studies have examined the risks for stroke in patients with MS, but questions remain about the findings. MS patients had a greater likelihood of being hospitalized for ischemic stroke (odds ratio (OR): 1.66, 95 % CI = 1.33–2.09) when compared to non-MS controls [36], but there were no differences or elevated risk for hemorrhagic stroke [36, 37]. A study on 32 different immune-mediated diseases (including MS) found that the risk for ischemic stroke was significantly increased (standardized incidence ratio = 3.05) in the year following a hospitalization for MS [37]. A study of 13,963 patients with MS compared to 66,407 non-MS controls from the Danish National Registry of Patients [17] also found a heightened risk of stroke shortly after MS diagnosis (for example, within a year) with the elevated risk being most pronounced in younger MS patients and absent in older MS subjects (≥56 years) [17]. However, the opposite trend for the effect of age on the risk of strokes in patients with MS was observed in a Swedish study [18]; complicating the interpretation, it is unclear if the same types of strokes were evaluated in these studies. When considering these studies, however, it is important to recognize that patients who are having an MS relapse are often misdiagnosed as having a stroke, particularly early in the course of their disease. Thus, some studies showing elevated risks for stroke in patients with MS may be inaccurate and reflect misdiagnoses or an increase in the surveillance (for example, MRI and more frequent physician visits) of this patient population identifying asymptomatic lesions suggestive of strokes [38].

Venous thromboembolism (VTE) appeared to be elevated in patients with MS compared to controls [18, 19, 21–23]. Ocak and colleagues [39] found an increased risk of venous thrombosis in MS patients: the OR for venous thrombosis in MS was 2.4 (95 % CI 1.3–4.3), and the OR increased to 12.5 (95 % CI 1.5-107.9) in patients with both MS and increased factor VIII levels, which indicates the additional risk factor of thrombophilia. Also, APLAs, which are a diagnostic feature of APS that results in thromboses, occur in a greater percentage of MS patients compared to controls (discussed later in the subsection “ASA and antiphospholipid antibodies”).

Individuals who have an occupation that involves prolonged sedentary behavior or patients experiencing immobilization have an increased risk of venous thrombosis compared to more mobile people [39, 40]. A sedentary lifestyle is also associated with an increased risk of stroke [41]. The activity level of patients with MS is less than that of healthy control subjects [42]. Physical inactivity, particularly in patients needing a wheelchair or who are bedridden, may contribute to the higher prevalence of thrombosis and ischemic strokes in patients with MS [43, 44].

Whether patients with MS have an increased risk for myocardial infarction is unclear [20]. In one study, MS patients were found to have decreased risks of being hospitalized for myocardial infarction and ischemic heart disease [36]; however, a recent study conducted with an initial cohort of 8,281 MS patients in Sweden determined that the risks for myocardial infarction, heart failure, and stroke were increased in individuals with MS compared to matched controls, and that these risks were more pronounced in women than men [18]. Shared pathological factors (for example, inflammation, oxidative stress, thrombogenic factors) between MS and cardiovascular diseases may explain the association between these conditions [18]. Of note, MS patients with ≥ 1 cardiovascular risks had increased MRI indications of disease activity, that is, more brain atrophy and an increase in lesion burden [45].

Treatments for MS may also increase the risk for cardiovascular diseases [20]. For example, systemic glucocorticoids have been reported to increase risks of stroke, myocardial infarction, and atrial fibrillation [20]. Additionally, a positive association was found for cardiovascular risk factors and the use of disease modifying therapies (DMTs) such as interferon-β and glatiramer acetate [46]. For example, about 20 % of MS patients on DMTs versus about 5 % of MS patients naïve to DMTs had diastolic blood pressure above 90 mmHg, and about 25 % of MS patients on DMTs versus about 14 % MS patients naïve to DMTs had glucose > 100 mg/dL [46]. The association was more pronounced in chronic progressive MS patients compared to relapsing remitting MS (RRMS) patients, but the correlation to disease activity (for example, rate of clinical relapse) was weak [46]. Although similar studies have not been conducted for more recent DMTs, cardiac side effects can be associated with these agents. For example, an increase in hypertension has been noted for teriflunomide (4 % treated versus 2 % placebo [47]). A reduction of heart rate can occur within six hours of fingolimod initiation [48], and there can be an increase in blood pressure in a small percentage of patients over the long term [49].

### Increased risk of dying from cardiovascular disease in patients with MS

Besides a possibly increased risk of cardiovascular disease (CVD), studies generally suggest that MS patients have a heightened risk of dying from CVD [43, 50]. As reviewed in Christiansen [20], patients with MS have a 6–34 % greater risk of death by CVD or stroke than for the overall population, and greater than 10 % of MS patients may have stroke as the cause of death. An analysis of 9,881 MS patients in the Danish Multiple Sclerosis Registry revealed that the mortality rate from CVD was significantly higher in MS patients compared to matched controls [50], and an analysis of over 6,000 patient deaths in the Danish MS Registry revealed that 17.6 % of deaths were caused by vascular or cardiac diseases, which was the most frequently listed cause of death outside of MS itself [43]. The standard mortality rate (the number of subjects who died from the specific cause divided by the number of deaths expected from population mortality statistics) of cardiac or vascular diseases in this same patient population was 1.34, indicating about a 34 % greater chance of death by CVD in MS patients when compared to general population mortality statistics [43]. In contrast, a study in South Wales revealed that while CVD caused 16 % of deaths in the MS population surveyed, this rate did not differ from the expected death rates in the general population [51].

While it appears that patients with MS have a greater likelihood of dying from cardiovascular-related issues, the underlying causative reason is not clear. These patients may lead a more sedentary lifestyle than healthy individuals due to motor symptoms and fatigue, for example [42], particularly as the disease progresses, and a less active lifestyle has been associated with cardiovascular risk factors such as impaired glucose tolerance and the development of metabolic syndrome in the general population [52, 53]. Furthermore, altered metabolic responses in patients with MS, such as increased adipose lipolytic activity, could be a factor in lower physical performance [54]. Several studies (reviewed in [55]) suggest that patients with MS are more likely to develop vascular disease and comorbidities related to metabolic syndrome (such as obesity, impaired glucose tolerance, and dyslipidemia), but these factors may develop or worsen as a result of progressively debilitating MS symptoms. Because many of these studies are retrospective, correlational designs, the exact cause and effect relationship between MS and CVD risk and mortality cannot be easily discerned. It is unclear whether CVD risk factors contribute to MS disease pathogenesis, whether MS symptoms promote the development of CVD and mortality, or whether a shared underlying disturbance, such as in glucose metabolism [56], underlies both disease processes.

## Treatment potential of ASA for cardiovascular disease in patients with MS

### ASA mechanisms of action

**Fig. 1:**
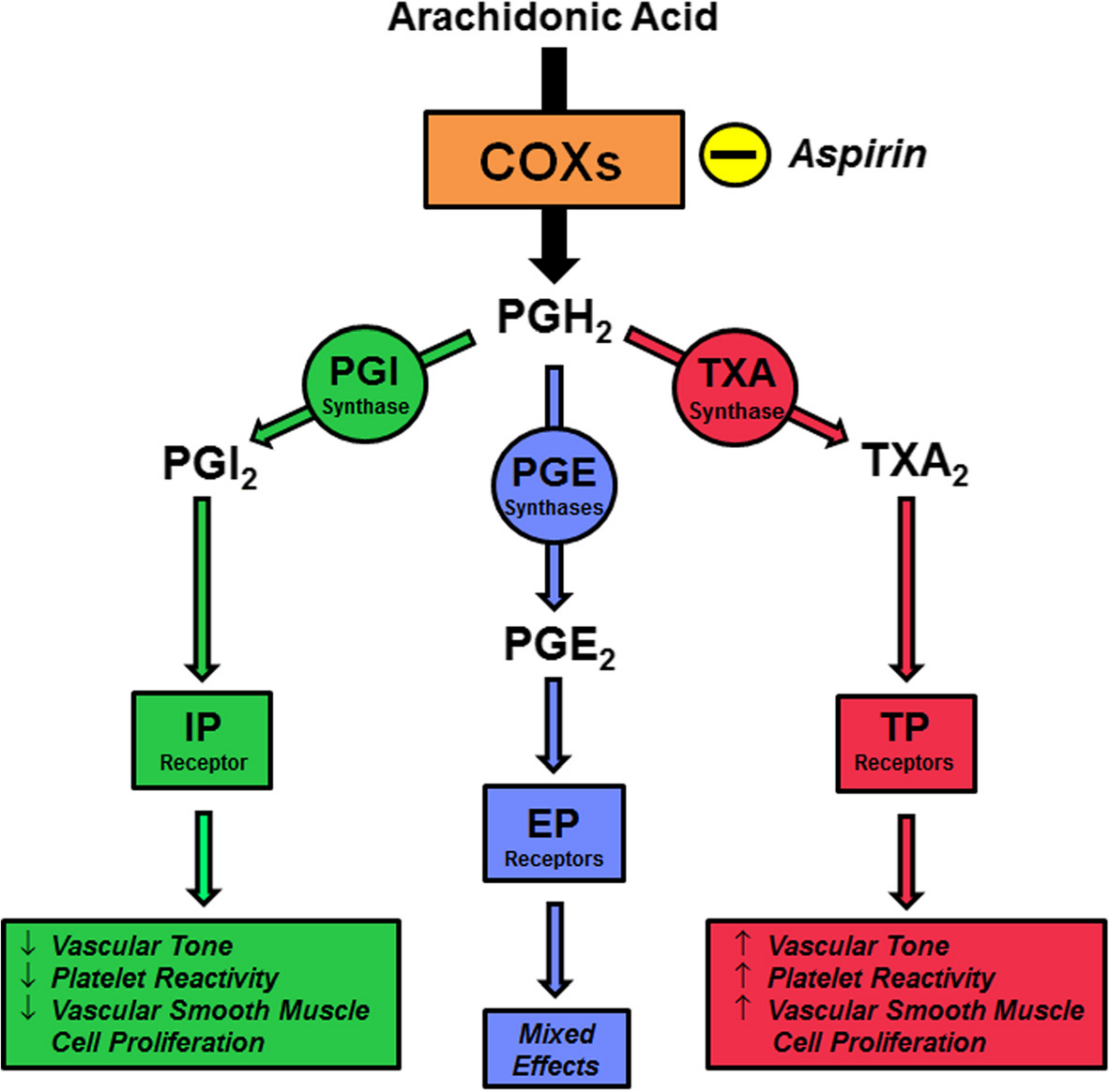
Aspirin inhibition of the synthetic pathway of prostaglandins I_2_, E_2_, and thromboxane A_2_. Cyclooxygenases metabolize arachidonic acid to PGH_2_, which in turn is converted into various prostanoids by specific enzymes. Depending on the receptors activated by these molecules, mixed physiologic effects on the vasculature and platelet reactivity occur. Aspirin irreversibly inhibits cyclooxygenase activity. COXs = cyclooxygenases; PGH_2_ = prostaglandin H_2_; PGI synthase = prostaglandin-I synthase; PGE synthases = prostaglandin-E synthases; TXA synthase = thromboxane synthase; PGI_2_ = prostacyclin; PGE_2_ = prostaglandin E_2_; TXA_2_ = thromboxane A_2_; IP receptor = prostacyclin receptor; EP receptors = prostaglandin E_2_ receptors; TP receptors = thromboxane A_2_ receptors

Aspirin (ASA) is a traditional nonsteroidal anti-inflammatory drug (tNSAID) used to treat inflammation, pain, and fever, and to inhibit platelet activation and aggregation. This latter effect inhibits thrombus formation, thus providing cardioprotection, and is the basis for the use of ASA in the prevention of myocardial infarction [31, 57, 58]. The many therapeutic uses of ASA are due to its inhibition of cyclooxygenases (COXs), enzymes that catalyze a step in the production of prostanoids (Fig. 1). Prostanoids include prostaglandins, prostacyclin, and thromboxanes, molecules with pleiotropic effects on a large number of physiologic systems [59].

A unique quality of ASA that differentiates it from other tNSAIDs is its ability to covalently acetylate COXs [60], whereas other tNSAIDS are competitive, reversible inhibitors [59, 61]. The irreversible linkage by ASA inhibits the enzyme’s ability to convert arachidonic acid to prostaglandin H_2_ (PGH_2_), a committed step in prostanoid synthesis, and recovery of the system is directly related to production of new COX enzymes. Cyclooxygenase-1 (COX-1) is considered to be constitutively expressed and can be identified in most tissues [62]. Cyclooxygenase-2 (COX-2) is generally considered an inducible isozyme found in monocytes, endothelial cells, and fibroblasts, although it is also constitutively expressed in some cells within the brain, testes, and kidney [62]. Therefore, COX-1 is thought to be the dominant source of prostanoids for housekeeping functions and COX-2 is considered to be a main source in inflammation, although platelet-derived prostanoids generated through COX-1 are linked to promotion of an inflammatory state [59, 63–65].

Once formed, PGH_2_ may be converted to prostaglandin E_2_ (PGE_2_) by prostaglandin-E synthase; prostacyclin (PGI_2_) by prostaglandin-I synthase; or thromboxane A_2_ (TXA_2_) by thromboxane synthase [66, 67]. These mediators interact with specific G-protein coupled receptors that utilize either cAMP or IP_3_/DAG/Ca^2+^ as second messengers ultimately to elicit physiologic responses that often are in opposition to each other. In terms of vascular smooth muscle tone and platelet reactivity, PGI_2_ and TXA_2_ act conversely, with PGI_2_ decreasing and TXA_2_ increasing these parameters [64]. Also, PGI_2_ inhibits vascular smooth muscle cell proliferation and TXA_2_ promotes it [64]. Increases or decreases in vascular tone and platelet reactivity can be elicited by PGE_2_, depending on which of its five receptors are activated [64].

Platelets participate in the thrombus formation process, and as such, hyperactivity built upon underlying atherosclerosis can contribute to the development of pathological cardiovascular events that may result in decreased blood flow, acute coronary syndrome, and stroke [68, 69]. Through irreversible inhibition of platelet COX-1, and the resultant decrease in TXA_2_ production, ASA decreases platelet activation and aggregation, and demonstrates a cardioprotective effect [57, 64]. Inflammation plays a role in the development of atherosclerotic plaques, and evidence suggests that platelets contribute to inflammatory processes through activation of vascular endothelial cells and leukocytes in inflamed microvessels [70]. Furthermore, platelets contain dense granules and α-granules that store preformed chemical mediators. Upon platelet activation and subsequent degranulation, these mediators, which include growth factors, cytokines, and coagulation factors, are released and can allow the platelet to influence the vascular endothelium and the inflammatory response [70, 71]. In neuroinflammatory disease states like MS, platelet activation could aggravate the disease process [65, 71–73]. In this regard, ASA inhibition of platelet COX-1 could potentially limit production of proinflammatory eicosanoids and attenuate the inflammatory state.

Since the COX inhibition by ASA is irreversible, the antiplatelet effect is dependent on the synthesis of new platelets. Therefore, lower doses of ASA (50–100 mg/day) are effective due to action within platelets, thus making it useful in the treatment of coronary artery disease [31, 57, 58, 64, 74]. Distinct from this platelet effect, higher doses are used for analgesic, antipyretic, and anti-inflammatory effects and are usually taken only as needed as opposed to daily [59].

### ASA use in preventing cardiovascular disease

In order to assess the potential impact of ASA to counter cardiovascular events in MS, it is helpful to address its role in reducing CVD in other segments of the population. In the general population, regular ASA use for prevention of cardiovascular events ranges between 18–41 %, and usage can be more prevalent in subpopulations, for example, diabetics and older individuals who are at higher risk for CVD [75–78]. Long-term administration of ASA at low doses helps prevent strokes, heart attacks, and blood clot formation in people at high risk of these events [24–26]. In an analysis of antiplatelet treatment following acute ischemic stroke, Sandercock et al. [79] found a significant decrease in recurrent ischemic stroke and death in patients who began ASA treatment no more than 14 days following a presumed ischemic stroke occurrence (OR 0.95, 95 % CI 0.91–0.99). It has also been noted that although there is a slight increase in the incidence of intracranial hemorrhages with antiplatelet treatment, the benefits of preventing repeat ischemic strokes and other cardiovascular events such as pulmonary embolism (PE) outweigh the risks of intracranial bleeding [30, 79]. ASA monotherapy (50–325 mg/day), clopidogrel, or extended-release dipyridamole (ER-DP) combined with ASA are therapies recommended by the American Heart Association and American Stroke Association, as well as the Eighth American College of Chest Physicians (ACCP), for the secondary prevention of ischemic strokes in individuals with a history of ischemic events including stroke [25, 80, 81]. The combination of ASA and ER-DP is preferable to ASA alone, while the combination of ASA with clopidogrel has a heightened risk of bleeding events, and thus should be avoided [25, 26, 80–83].

Although ASA has been established as effective for preventing secondary cerebrovascular events [26], its prophylactic efficacy is less clear. A significant reduction in the risks for a first myocardial infarction was found with ASA usage, but results for ASA’s efficacy in preventing a first stroke and CVD were inconclusive [31, 84]. A review of 27 studies on the effectiveness of ASA for the primary prevention of cardiovascular events (for example, in patients not at risk for CVD) revealed only modest/minor benefits that did not outweigh the risks of increased bleeding and hemorrhagic strokes [32].

Platelets were traditionally considered to play a greater role in arterial rather than venous thrombosis, but platelets have been shown to have a role in venous thromboembolism (VTE) by inducing the formation of neutrophil extracellular traps, releasing proinflammatory mediators and microparticles, and aggregating as a component of thrombi themselves [27]. While some studies such as the Longitudinal Investigation on Thromboembolism Etiology did not find a reduction in VTE by ASA users [85], other trials report that ASA lowers the risk of deep vein thrombosis (DVT) [29]. The INSPIRE study combined data from two previous studies where patients were given ASA after a first unprovoked VTE to determine its effects on VTE recurrence. The INSPIRE analysis found that ASA reduces the overall risk of recurrent VTE by 42 % (*P* = 0.005) with only minor bleeding concerns, supporting the use of ASA for secondary prophylaxis of VTE [28]. In the large Pulmonary Embolism Prevention (PEP) trial, which included over 13,000 patients undergoing surgery for hip fracture or elective arthroplasty, PE or DVT was experienced in 105 out of 6,679 (1.5 %) patients receiving 160 mg ASA compared to 165 of 6,677 (2.5 %) patients receiving a placebo, which equals a proportional reduction of 36 % PE/DVT in those treated with ASA (*P* = 0.0003) [86]. ASA was also found to be effective for the prevention of PE in high-risk patients by the Antithrombotic Trialists’ Collaboration in a 2002 meta-analysis: patients taking an antiplatelet (for example, ASA) had a 25 % reduced risk of acute PE compared with patients on placebo (*P* < 0.01) [87]. Based on results such as these and the low cost and relatively low risk of bleeding of ASA when compared to warfarin and newer anticoagulants, the American Academy of Orthopedic Surgeons in 2009 and the ACCP in 2012 included ASA as a method in their guidelines to prevent VTE in high-risk orthopedic surgery patients [27, 88].

### Considerations regarding ASA use for cardiovascular disease in patients with MS

Given that ASA may reduce cardiovascular events in non-MS patients at risk, the possible elevated risks for stroke and venous thrombosis in MS, along with the observance of vessel congestion [14, 89–91] and altered perfusion of cerebral structures [9–12, 92] in this population, suggests that MS patients taking ASA may have some degree of protection against stroke or venous thrombosis (Table 1). However, the slight risk of increased intracerebral bleeding observed in stroke patients given ASA [79] is troubling, since MS patients have a compromised BBB [7, 8] and ASA treatment could further disrupt the BBB (Table 2). For example, the elevated coagulation proteins observed in the CNS of patients with MS [92] could be performing a protective role by limiting BBB leakage, and if ASA disrupted their deposition, then this could slow the resolution of the leakage. However, ASA can act to limit vascular leakage: ASA reduced arachidonate-induced vascular leakage in the peritoneum [93] and induced lipoxin A_4_, which was found to reduce vascular leakage following acute ear inflammation [94]. ASA also lowered the permeability of the BBB in non-stroke patients [95].

It might be important to identify patients with MS who have a heightened risk of a cardiovascular event, as they would be expected to have the most favorable risk-to-benefit ratio when using ASA. Besides the traditional risk factors for cardiovascular events, there are features of MS that may potentially lead to increased risk of CVD within the MS population. For example, a more sedentary or immobile state in MS patients [42, 44] or taking an MS DMT [46–49] might be associated with an increased risk of cardiovascular events.

### ASA and antiphospholipid antibodies

Multiple studies have reported that APLAs are more prevalent in patients with MS than in the general population [96–98]. APLAs are the main diagnostic feature of APS, an autoimmune disease characterized by thrombosis and/or pregnancy morbidity and elevated levels of anticardiolipin antibodies, lupus anticoagulant, and/or β_2_ glycoprotein I [99]. MS and APS share various features, and one disorder can sometimes be misdiagnosed for the other [96, 99]. Although the role that APLAs may have in MS is presently unclear, recent studies indicate a relationship between APLAs and a more severe MS disease course [97, 98, 100–102]. In a prospective three-year study following interferon-β (INF-β)-treated MS patients with or without APLAs, Zivanidov et al. found that the APLA-positive patients showed a greater disease progression as measured by higher MRI lesion volumes, increased tissue damage, and loss of brain volume, as well as more clinical relapses [102]. The presence of anti-INF-β binding antibodies may decrease the efficacy of INF-β treatment, and Garg et al. [103] found a significant co-occurrence of high APLAs in MS patients with anti-INF-β binding antibodies. A higher frequency of APLAs has been reported in secondary progressive MS (SPMS) compared to RRMS, which is consistent with the idea that the presence of APLAs is related to a more chronic, advanced stage of the disease [97, 102, 104].

Given that thromboses are a main symptom in APS, anticoagulation, antiaggregation, and ASA are all used as treatment for this syndrome [96, 99, 102]. Although many physicians prescribe daily low-dose ASA for asymptomatic APLA-positive patients to prevent a first thrombotic event, results from studies on the benefit of ASA prophylaxis in this population are mixed [105]. The Antiphospholipid Antibody Acetylsalicylic Acid study found that 81 mg of ASA daily for APLA-positive but asymptomatic patients was not more effective than placebo in protecting against a thrombotic event [106]. In contrast, in an individual patient-level meta-analysis from five international cohort studies, Arnaud and colleagues [107] found that prophylactic low-dose ASA in patients with APLAs significantly reduced the risk of a first thrombotic event. Although the role of APLAs in the pathogenesis of MS is not clear, given that MS is associated with an increased risk of thrombosis and cardiovascular events (see, for example, [18, 20, 21]), MS patients with APLAs may benefit from anticoagulation or ASA treatment.

### Potential effects of ASA on vascular pathology in MS

In addition to affecting thrombosis and stroke in MS patients, ASA could affect other components of vascular pathology in MS. Pathological changes to the vascular tissue in the CNS have been observed since the earliest descriptions of MS (see, for example, [89, 108]), and they include platelet and fibrin deposits associated with vessels in active lesions, vessel occlusion, vascular thickening, enhanced deposition of perivascular collagen, BBB disruption, and perivascular inflammation [7, 8, 14–16, 109]. Proteomic analysis revealed the deposition of an array of coagulation proteins in chronic active plaques [110], and anticoagulants decreased the severity of experimental autoimmune encephalomyelitis (EAE) [110], an animal model of MS, indicating that vascular changes have a detrimental role in the disease.

Platelets are activated in MS and have been implicated in contributing to pathogenesis, for example, by promoting inflammation [65, 71–73]. Platelets were identified in MS and EAE CNS lesions, and depletion of platelets lessened EAE severity [73]. Two indicators of platelet activation, β-thromboglobulin (β-TG) and platelet factor 4 (PF4), were higher in the plasma of MS patients during quiescent disease compared to control subjects (*P* < 0.001) [111]. ASA 50 mg/day taken orally by patients with MS significantly lowered plasma β-TG levels (*P* < 0.001), but not to normal levels [111]. PF4 levels did not undergo a significant decline following ASA treatment, suggesting that a source other than platelets, for example, mast cells, could have been an important contributor to PF4 levels in the plasma of these patients [111]. In normal male subjects, ASA decreased platelet aggregation and P-selectin expression in a dose-dependent manner, and low ASA doses increased the dilatation of the brachial artery while high doses decreased flow [112]. Although these studies suggest a benefit of ASA by inhibiting platelet activation, more studies are needed to confirm the results and elucidate the specific mechanism of action.

Besides platelets acting to promote inflammation in EAE [73], fibrin deposition has been credited with activation of microglia, which have been associated with tissue damage [113]. Depending on the type, microglia can have different influences on disease activity in MS; that is, they can induce tissue damage or promote repair [5]. Blockage of fibrin formation lessened disease activity in EAE [114, 115]. ASA may promote fibrinolysis or interfere with fibrin deposition [116].

In addition to fibrin, thrombin activity has been associated with worsening of inflammatory CNS disease states such as MS [117], and thrombin activity in the spinal cord of mice with EAE is associated with multiple pathological features [118]. ASA may decrease thrombin levels at sites of microvascular injury [119]. In experimental APS, which models a condition that results in clots in deep veins and in organs such as the brain, ASA also reduced tumor necrosis factor alpha (TNFα) and prostaglandin E synthesis and increased thrombin inhibitors [120].

Vascular changes may contribute to the decreased perfusion observed in MS [9–12, 92, 121]. Decreased perfusion has been observed in the cortex [9, 10, 12], deep gray matter structures (for example, thalamus, caudate) [10–12], and normal-appearing white matter (NAWM) [92] of patients with MS. In addition, white matter lesions from patients with RRMS had altered perfusion (some with decreased perfusion and others with an increased perfusion compared to that from white matter in control subjects) [10, 122]. The extent of white matter lesions was correlated with decreased cortical blood flow [123], and decreased cerebral blood flow has also been observed in EAE [124, 125].

Decreased perfusion could impair tissue oxygenation. Decreased tissue oxygenation in MS patients has been detected in white matter and cortex gray matter by positron emission tomography [126], and decreased utilization of oxygen in MS was revealed in periventricular veins by susceptibility-weighted imaging (SWI) [127] and by T2-relaxation-under-spin-tagging of venous sinus blood [128]. Additionally, lesions observed by SWI in the spinal cord of EAE mice were thought to be detected largely due to deoxyhemoglobin, whose presence was likely a result of hypoxia [129]. At the molecular level, upregulation of hypoxia-inducible factor-1α (HIF-1α) has been observed in active MS plaques by immunohistochemistry [130, 131], and the upregulation of genes associated with ischemic preconditioning, including HIF-1α, has been observed in NAWM in patients with MS [132]. Additionally, endoplasmic reticulum stress proteins, which have been associated with ischemic injury, are overexpressed in active MS lesions [133, 134] and in gray matter MS lesions [135]. Although altered perfusion may reduce oxygen delivery and impair energy production [136], vascular changes, that is, microvascular thrombosis, as the cause of hypoxia-like changes in MS have been questioned, since hypoxia-like changes have been observed in MS patients in the absence of vascular pathology [137].

Mitochondrial dysfunction has been observed in multiple sites in the CNS of MS patients, including NAWM, lesions, and cortex, and altered mitochondria function may help create a hypoxic state [35, 137–139]. ASA may act to inhibit complex I of the respiratory chain [33]. Complex I is decreased in chronic active white matter lesions and in the motor cortex of patients with MS [34, 35]. Thus, ASA has the potential to further lower complex I activity in MS, and ASA’s inhibitory effect may be more pronounced with depletion of glutathione [140], which is thought to occur in patients with MS [141, 142]. Furthermore, inhibition of complex I activity can lead to an increased production of reactive oxygen species (ROS) [143], which could amplify cellular damage.

Cerebrovascular reactivity, the ability of the cerebral vasculature to increase local blood flow via arteriole dilation in response to neural activity, is impaired in MS, perhaps as a result of vascular desensitization from chronic high levels of nitric oxide stemming from inflammation [144]. Interestingly, ASA has been found to increase endothelial nitric oxide synthase activity [145, 146], which would favor an increase in blood flow, but not if vascular desensitization has developed [144].

## ASA effects on MS symptoms

### ASA in MS, EAE, and related studies

The cardiovascular effects of ASA would not be expected to readily translate into gross alterations of MS disease activity. Only a couple of studies have looked directly at the effects of ASA on MS disease activity, and they were performed decades ago using relatively elementary outcome measures. Two studies in the early 1960s examined the effects of calcium aspirin (Solprin) (3.5 g divided over 3 doses per day) against prednisolone (15 mg once per day initially; reduced to 10 mg per day at 8 months) and placebo (lactate tablets) [147], or chloroquine (250 mg once per day) and placebo [148], in MS. Each patient’s disease condition was measured using Alexander’s 1958 numerical scoring system prior to treatment and at 6 and 18 or 6 and 14 months following treatment, respectively. No significant differences were found between treatment groups in either study, although patients receiving Solprin in the prednisolone study deteriorated the least over 18 months [147], and Solprin performed better than chloroquine but worse than the placebo in the latter experiment [148]. Given that these studies were conducted using an outdated measure of MS disease activity, the interpretation of the results should be viewed cautiously. MRI studies, including those examining the vasculature and blood flow, and other more current measures of MS disease activity could provide greater insights regarding the effect of ASA on disease progression.

Studies examining ASA in animal models of MS also have been relatively sparse. One study published in 1949 examined the prophylactic and therapeutic effects of sodium salicylate (the main metabolite of ASA) and para-aminobenzoic acid, either alone or in combination, in a guinea pig EAE model [149]. Although neither compound showed effects against disease onset and progression at moderate dosage levels, the combination of sodium salicylate and para-aminobenzoic acid, as well as larger doses of sodium salicylate by itself, seemed to delay onset, lessen incidence, and inhibit disease severity if administration was begun prior to or shortly after (5 days or less) EAE induction [149]. Treatment with sodium salicylate or the combined drugs after the animals became sick had no effect on the disease [149]. When ASA was tested in the guinea pig EAE model by another group, there was no beneficial effect [150]. In the Lewis rat model of EAE, ASA delayed the onset of disease but increased the severity of disease [151], while sodium salicylate postponed disease onset and reduced clinical signs [152].

More recent studies have looked at the role of COX-1 and COX-2 in EAE. Naproxen, a COX-1 and COX-2 inhibitor, was shown to delay EAE onset and reduce the severity of the disease when treatment was started on the day of EAE immunization [153]. In another study, celecoxib, a new generation COX-2 inhibitor, reduced EAE incidence and/or severity when animals were treated beginning on the day of EAE induction or 8 days post-induction [154]. But celecoxib also reduced disease severity in COX-2-deficient mice; and nimesulide, another COX-2 inhibitor, did not affect disease development or severity, which indicates that the mechanism of action of celecoxib on EAE is not via the COX-2 pathway [154].

ASA was found to limit the production of ROS and proinflammatory cytokines (for example, TNFα and IL-1β) by a microglial cell line treated with the activator lipopolysaccharide [155, 156], and ASA limited proinflammatory cytokine production and microglial activation following middle cerebral arterial occlusion in the rat [157]. Activated microglia can mediate tissue damage in MS [3–5], and lessening their production of inflammatory mediators by ASA could have possible benefits. Also, it may be that ASA could promote resolution of inflammation in MS by inducing lipoxin A_4_ [158], which is an anti-inflammatory mediator [159].

In salt-loaded, stroke-prone, spontaneously hypertensive rats, ASA suppressed BBB damage and reduced several markers of inflammation (for example, matrix metalloproteinase-9 activity, superoxide production, and macrophage accumulation) [160], raising the possibility that ASA could limit similar pathological processes in MS.

Recent studies found that ASA upregulated the production of the ciliary neurotrophic factor [161], which augments myelin formation [162]. ASA also induced the proliferation and differentiation of oligodendrocyte precursors and limited demyelination following a cerebral ischemic lesion [163]. If ASA acted similarly in response to MS lesions, then it could promote remyelination efforts [161].

### ASA and fatigue

Fatigue is a pervasive and debilitating symptom associated with a marked decrease in the quality of life for patients with MS. The cause of fatigue is not understood. Some possible theories concerning the causes of fatigue have included: elevated body temperature (in RRMS patients) [164], sleep disturbances and depression [165, 166], proinflammatory cytokines [167], reduced metabolism and degeneration of cerebral and deep gray matter structures [168, 169], reduced connectivity [170], and reduced perfusion of deep gray matter [11]. There are a limited number of approaches used to counter fatigue in MS, for example, amantadine, modafinil, vitamin D analog, treatment for sleep disorder, and exercise [171–175]. Overall, studies have been conflicting as to the benefits of these modalities, and better management of fatigue is sorely needed.

ASA has been tested as a way to counter fatigue (Table 1). Following observations in a clinical setting that some MS patients seemed to experience a lessening of fatigue while taking ASA for non-MS-related symptoms, a double-blind, randomized, crossover study of 650 mg oral ASA twice daily or placebo for 6 weeks was initiated, where a modest but detectable improvement was found during the treatment phase with ASA [176]. The mean obtained from the Modified Fatigue Impact Scale (MFIS, range 0–84), which was administered weekly, decreased from 46.3 ± 16.0 at baseline to 38.1 ± 17.0 during ASA administration versus 42.5 ± 18.8 during placebo (ASA versus placebo, *P* = 0.043) [176]. In addition, of the patients completing the crossover components of the study, only 1/26 patients preferred placebo compared to 10/26 of patients preferring ASA treatment (*P* = 0.012) when responding to the Global Fatigue Change self-assessment [176]. None of the other outcome measures for fatigue assessment revealed statistically significant differences (10-point Visual Analog Scale, Fatigue Severity Scale [FSS], MS-Specific Fatigue Scale), but there was a trend towards a greater reduction of fatigue symptoms while taking ASA on the Visual Analog Scale (ASA versus placebo, *P* = 0.076) [176].

A subsequent randomized double-blind crossover clinical trial for 52 patients with MS was conducted over a ten-week period to study the effectiveness of ASA and amantadine for alleviating fatigue in MS. Half of the patients were randomly assigned to receive 500 mg ASA orally once daily for the first four weeks, and following a two-week washout period were switched to 100 mg amantadine orally twice a day for the final four weeks; the other half of the patients received the treatments in reverse order [177]. A significant decrease in self-reported fatigue levels measured using the FSS with both ASA and amantadine was found following a baseline measurement [177]. After the first round of treatment, mean FSS scores decreased by 1.1 (from a maximum of 7) for ASA and by 0.8 for amantadine [177]. During the two-week washout period, the mean FSS scores increased back to baseline levels [177]. In the second phase of the study, where patients received crossover treatments, self-reported fatigue scores were once again reduced significantly for each treatment regimen: mean FSS decreased by 0.7 for ASA and by 1.6 for amantadine [177]. The authors noted that both ASA and amantadine were well-tolerated by patients with few and mild side effects, none of which led to participant dropouts. Given the promising results shown by both treatments in these studies, they suggest that ASA and amantadine deserve further consideration as potential treatments to combat fatigue in MS [177].

A placebo-controlled, double-blind, multicenter study was performed comparing placebo, 162 mg/d and 1,300 mg/d of ASA [178]. Although the study was not completed, an intermediate analysis of the placebo and high dose groups revealed a difference of 4.6 points on the MFIS, that is, adjusted mean scores of 42.7 versus 38.1 in the respective groups. However, the study was underpowered and it did not reveal a statistically significant effect [178]. The authors indicated that it is unlikely that ASA provides a clinically relevant benefit for MS patients [178]. It is possible that confounding factors, such as an undiagnosed sleep disorder [166], could interfere with ASA effect on fatigue.

The mechanism of action by which ASA might lessen fatigue, even minimally, is unclear. ASA’s reduction of fatigue symptoms may be through the drug’s antipyretic effects [176, 177], which is supported by a recent study that found a correlation between fatigue, as measured by FSS and MFIS, and elevated body temperature in RRMS patients [164]. In addition, ASA could have affected other systems (for example, autonomic or neuroendocrine) involved in the perception of fatigue [176, 177]. ASA also could have countered proinflammatory cytokines [176, 177], which may contribute to fatigue [167, 179]. ASA may also act on fatigue associated with some form of interferon-β therapy, which was taken by 5/30 patients in the Wingerchuck et al. (2005) study [176] and by 52/52 patients in the Shaygannejad et al. (2012) study [177].

### ASA and depression

Patients with MS are more likely to experience depression than the general population [180]. Although depression has traditionally been thought of as a neurotransmitter-related/driven disease, evidence suggests that inflammation may play a role in the disorder [181]. As such, drugs that reduce inflammation may be beneficial in depression [181].

The use of ASA may lower the risk of major depression [182]. A study on depression and anxiety in patients with myocardial infarction found that those taking ASA reported fewer depression and anxiety symptoms (*P* < 0.01) as measured by the Hamilton Depression and Hamilton Anxiety Rating Scales, respectively [183]; and an analysis of 174 male coronary angiography patients (99 on ASA) found fewer depressive symptoms in those taking ASA regularly (range from 80 mg every other day to 325 mg daily), both by self-report (*P* = 0.016) and reported perceptions from a significant other (*P* = 0.048) [184].

In an established rodent model of depression, ASA lessened immobility in a forced swim test in rats and concurrently attenuated cytokine levels (IL-6 and TNF-α) [185]. Additionally, preliminary clinical trials have reported that ASA in combination with antidepressants (fluoxetine) can shorten the onset of antidepressant action and be effective against treatment-resistant depression [186], and this effect has also been demonstrated in rats [187, 188]. However, 8 participants in a small clinical sample (10 total) of patients experiencing depression who were treated with 160 mg/day ASA in combination with 20 mg/day of the antidepressant citalopram experienced severe side effects (anxiety and akathesia) that necessitated the hospitalization of 4 participants while 2 other patients exhibited suicidal behavior, resulting in the termination of the study at 14 days [189]. As such, the authors caution the use of ASA in combination with certain antidepressants such as citalopram [189]. Furthermore, NSAIDs such as ibuprofen and ASA may interfere with the antidepressant effects of selective serotonin reuptake inhibitors (SSRIs) [190]. It has also been found that the combined treatment of ASA with an antidepressant (SSRI) increases the risk of bleeding over ASA alone in patients who had experienced an acute myocardial infarction [191]. Because damage of the BBB [7, 8] and microhemorrhages [192–195] occur in MS, caution is warranted when adding ASA to the treatment regimen for MS patients who may be concurrently receiving antidepressant treatment for depressive symptoms.

## Potential risks associated with ASA use in MS

Due to the known pleiotropic effects of the prostaglandins and thromboxanes on multiple physiological systems, it is not unexpected that inhibition of their production by ASA could potentially cause various adverse effects. Many of the major adverse effects related to ASA use are discussed below and summarized in Table 2.

While the incidence of cerebral hemorrhage during ASA treatment is low in most studies, the antiplatelet effect of ASA can contribute to the development of increased cerebral bleeding [30]. The Antithrombotic Trialists’ Collaboration performed a meta-analysis (6 primary prevention trials and 16 secondary prevention trials) of serious vascular events, including stroke and major bleeds comparing long-term ASA versus control [31]. Their conclusions included that ASA increased incidences of hemorrhagic stroke in both primary (*P* = 0.05) and secondary (*P* = 0.07) prevention trials and when analyzed in combination (*P* = 0.01), while ASA showed a protective effect concerning ischemic stroke (*P* = 0.005) [31]. Sutcliffe et al. [32] reviewed data from randomized trials assessing ASA in the primary prevention of CVD and cancer and concluded that the benefits of ASA for primary prevention of CVD are modest, and are much less than those for secondary prevention. Furthermore, while benefits and harms were low based on person-years, they estimated an increased risk of hemorrhagic stroke ranging from 32–38 % [32].

Although the increases in incidents of hemorrhagic stroke noted are slight, it is possible that in disease states like MS, where the BBB is disrupted or compromised [7, 8], the risk of intracerebral bleeding may be greater with ASA usage. Additionally, there is an increased risk of immune thrombocytopenia in patients with MS [196], which would be a counter indication of ASA usage due to the enhanced risk of bleeding with ASA in this patient population [197].

An adverse effect that is well associated with ASA use is upper gastrointestinal (GI) tract injury and bleeding. Because ASA inhibits the production of prostaglandins by GI-located COX-1, these gastroprotective substances are unavailable and damage may occur. The risk of complications is increased with aging, concomitant use of anticoagulants, history of NSAID-associated bleeding, and comorbidities [198]. The Antithrombotic Trialists’ Collaboration study noted above determined that ASA usage increased major GI and extracranial bleeds to 0.10 % versus 0.07 % per year in controls (RR = 1.54 [1.30–1.82], *P* < 0.0001) [31].

ASA and salicylate, the active metabolite of ASA, are known to cause hearing loss and tinnitus at high doses (for example, 6–8 g/day) [199–203]. The mechanisms behind these effects are unclear, but salicylate seems to have effects centrally on GABAergic neurotransmission [203, 204], as well as more peripherally by affecting cochlear function [200, 204]. Moreover, a strong linear relationship exists between unbound salicylate plasma concentration and a resultant decrease in auditory sensitivity [205], and the salicylate toxicity model is used by auditory scientists to investigate mechanisms underlying tinnitus [204].

Certain asthmatic patients have a sensitivity to ASA that manifests itself as a respiratory/asthma-type attack. Jenkins et al. performed a systematic review and found that the pooled incidence of ASA-induced asthma was 21 % in adults and 5 % in children [206]. This is higher than the value of approximately 10 % that has been published elsewhere [207, 208]. The mechanism of this reaction in ASA sensitive-individuals is thought to occur due to COX inhibition resulting in decreased PGE_2_ and thus unabated activation of the 5-lipoxygenase pathway. This, in turn, increases production of leukotrienes and mast cell release of histamine, leading to airway hyperreactivity [209, 210].

Prostaglandin-mediated vasodilation is necessary for proper renal plasma flow, especially in individuals with underlying renal disease, congestive heart failure, or cirrhosis. Through inhibition of renal COX-2, ASA and other NSAIDs can cause volume-dependent renal failure and that resulting from interstitial nephritis and nephritic syndrome [198].

## Conclusion

Although ASA use is relatively common in the general population, ASA usage by MS patients has the potential for positive and/or negative influences on different facets of the disease, that is, symptoms, disease mechanisms, and associated disease risks. Understanding the impact of ASA use on these features would help to further establish the risk-to-benefit ratio of ASA usage in this patient population. Venous thrombosis and possibly stroke have an elevated likelihood in MS [17–23, 37], and given that ASA can lessen the risks of these cerebrovascular diseases [25, 26, 29], it is likely that ASA confers a similar benefit of lower risk for MS patients. Fatigue is a relatively common symptom in MS, and ASA may ameliorate fatigue in MS patients [176, 177], although the effect size might be small [178]. ASA could also impact pathogenic processes. For example, ASA could act to reduce inflammation by limiting the production of proinflammatory mediators from activated microglia [155–157] or by inducing the production of lipoxin A_4_, which acts to resolve inflammation [158, 159]. Since a pathogenic role has been attributed to platelets and thrombin [65, 71–73, 117, 118], lessening their activation by ASA could potentially reduce their ability to stimulate inflammation or impair blood flow [112, 116, 119]. Despite these potential benefits, ASA usage can have negative side effects such as being associated with an elevated risk of hemorrhagic stroke [30–32]. ASA may also worsen some specific components of MS pathology, for example, enhancing leakage of the BBB and possibly inhibiting mitochondrial complex I activity [33], which is already reduced in MS [34, 35]. It is possible that subgroups of MS patients may find particular benefit from ASA, for example, immobile patients with increased risk for DVTs or patients with APLAs, which could counterbalance the risks associated with ASA usage. It is also possible that ASA usage has a small beneficial impact on overall disease progression. Thus, further studies are needed to determine the benefits and risks of ASA in patients with MS in order to establish proper guidance for ASA use by this patient population. Given the widespread usage of aspirin and the likelihood that many effects could be small, traditional placebo controlled trials would be unlikely to yield meaningful results. Carefully crafted population-based studies, while not definitive, may help guide our understanding of this complex issue.

## Abbreviations

ACCP: American College of Chest Physicians
APLA: antiphospholipid antibody
APS: antiphospholipid syndrome
ASA: aspirin
BBB: blood-brain barrier
CNS: central nervous system
COX-1: cyclooxygenase-1
COX-2: cyclooxygenase-2
COXs: cyclooxygenases
CVD: cardiovascular disease
DMT: disease modifying therapy
DVT: deep vein thrombosis
EAE: experimental autoimmune encephalomyelitis
ER-DP: extended-release dipyridamole
GI: gastrointestinal
HIF-1α: hypoxia-inducible factor-1α
MFIS: Modified Fatigue Impact Scale
MS: multiple sclerosis
NAWM: normal-appearing white matter
OR: odds ratio
PE: pulmonary embolism
PEP: Pulmonary Embolism Prevention (trial)
PF4: platelet factor 4
PGE synthase: prostaglandin-E synthase
PGE_2_: prostaglandin E_2_
PGH_2_: prostaglandin H_2_
PGI_2_: prostacyclin
ROS: reactive oxygen species
RRMS: relapsing remitting MS
SSRI: selective serotonin reuptake inhibitor
SWI: susceptibility-weighted imaging
tNSAID: traditional nonsteroidal anti-inflammatory drug
TXA_2_: thromboxane A_2_
VTE: venous thromboembolism
β-TG: β-thromboglobulin
